# “More Than My Own Effort”: A Qualitative Study of Factors Influencing EBP Competence Among Traditional Chinese Medicine Specialist Nurses

**DOI:** 10.1155/jonm/8658721

**Published:** 2026-06-24

**Authors:** Yan Li, Yu Chen, Wenji Xu, Jie Sun, Shizheng Du

**Affiliations:** ^1^ Department of Nursing, Jiangsu Province Hospital of Chinese Medicine, Affiliated Hospital of Nanjing University of Chinese Medicine, Nanjing, China, njucm.edu.cn; ^2^ School of Nursing, Nanjing University of Chinese Medicine, Nanjing, China, njucm.edu.cn

**Keywords:** COM-B model, competence, evidence-based practice, influencing factors, qualitative research, traditional Chinese medicine specialist nurse

## Abstract

**Aims:**

To explore factors influencing evidence‐based practice (EBP) competence among traditional Chinese medicine (TCM) specialist nurses using the Capability, Opportunity, Motivation, and Behavior (COM‐B) model.

**Background:**

EBP underpins quality and safety in nursing. TCM nursing is increasingly delivered across TCM and general hospital settings, yet integration of EBP within TCM nursing remains challenging. Little is known about what shapes EBP competence and practice among TCM specialist nurses who play key roles in delivering TCM nursing techniques and mentoring others.

**Methods:**

A qualitative study using semistructured interviews was conducted with 17 TCM specialist nurses between April and August 2024. Data were analyzed using theory‐informed thematic analysis. Initial coding was conducted inductively, and themes were subsequently refined and interpreted in relation to the COM‐B model.

**Results:**

Four overarching domains were identified and interpreted in relation to the COM‐B model: capability‐related factors (basic and advanced skills), opportunity‐related factors (stakeholder buy‐in, resource constraints, available time, and evidence‐related challenges), motivation‐related factors (role clarity and volitional tensions), and behavioral enactment of EBP competence in practice (inadequate engagement in EBP, self‐directed learning, and practice‐grounded expertise).

**Conclusions:**

Findings highlight the need for targeted strategies to enhance capability, expand institutional and systemic opportunities, address capability, opportunity, and motivation‐related barriers, and strengthen the enactment of EBP competence in practice. Such efforts may help strengthen the development and enactment of EBP competence among TCM specialist nurses.

## 1. Introduction

Evidence‐based practice (EBP) is a cornerstone of high‐quality healthcare and contemporary nursing, given its role in improving patient outcomes and informing clinical decision‐making. As traditional Chinese medicine (TCM) nursing continues to expand in areas such as chronic disease prevention and management, increasing attention has been directed toward strengthening its evidence base and promoting evidence‐informed practice. In this context, nurses’ competence in EBP is essential for the implementation of evidence‐based nursing (EBN), including within TCM nursing settings. In China, national policy has highlighted priorities related to strengthening EBN, advancing TCM nursing, and enhancing service capacity [1]. These developments underscore the need to build and sustain EBP competence within the TCM nursing workforce. Within this workforce, TCM specialist nurses may be particularly important because they are expected not only to deliver specialized TCM nursing care but also to support the uptake and dissemination of evidence‐informed practice in clinical settings [2].

TCM specialist nurses have expanded rapidly in mainland China and represent a key group for translating evidence into TCM nursing practice [3]. In China, TCM specialist nurses are generally registered nurses who have completed basic nursing education and registration, accumulated prior clinical experience, and then undertake post‐registration specialist training in TCM nursing [2]. Although training pathways vary across regions, programs typically combine theoretical instruction, supervised clinical practice, and competency assessment leading to certification, with training content commonly covering foundational TCM theories, syndrome differentiation–informed nursing, TCM nursing techniques, symptom management guided by TCM principles, and TCM‐informed patient education [2, 4]. After certification, TCM specialist nurses are expected to apply these competencies in clinical settings and increasingly support the delivery of evidence‐based TCM nursing [4–6].

For TCM specialist nurses, existing literature has predominantly documented the practice of TCM specialist nurses in TCM hospitals and nurse‐led clinics, with some reports also describing service delivery in community health institutions and home‐based services supported by internet‐enabled platforms [2, 6–8]. In addition, TCM nursing interventions have been reported as integrated into care pathways in general hospitals, for example, in stroke care, maternity care, and oncology symptom management [9–11]. However, these reports have focused primarily on service delivery models or the application of specific TCM nursing interventions, rather than providing clear descriptions of the responsibilities, role boundaries, and scope of practice of TCM specialist nurses in general hospitals. Because role expectations, service configurations, and practice demands may vary across settings, it remains uncertain whether factors identified in other nursing populations operate in the same way among TCM specialist nurses working across diverse practice contexts.

Despite increasing attention to EBP competence across nursing populations and specialties [12–15], evidence on TCM specialist nurses’ EBP competence remains limited. Prior studies among clinical nurses and nursing students have identified factors both at individual level (e.g., age, educational background, and beliefs about EBP) and contextual level (e.g., inadequate training, time constraints, lack of management support, and limited resources) [15, 16]. However, the direct transferability of these findings to TCM specialist nurses remains uncertain because the way in which these determinants are experienced and enacted may differ in TCM nursing contexts. TCM nursing practice is often guided by pattern‐based reasoning and individualized care, which may require nurses to integrate contemporary research evidence with syndrome differentiation and context‐sensitive clinical judgment [17]. In addition, what counts as “evidence” in TCM nursing may be understood more broadly, encompassing not only contemporary research but also traditional knowledge and practice experience [18]. Moreover, many TCM nursing interventions are complex and context‐dependent, with variability in components and outcome measurement, which may make evidence appraisal and implementation more challenging in practice [19, 20]. As a result, determinants such as training, resources, managerial support, and EBP beliefs may take different forms or function differently in TCM specialist nurses. These features may shape not only the practical use of evidence but also how EBP competence is developed, interpreted, and enacted by TCM specialist nurses. These considerations highlight the need to examine determinants of EBP competence within the specific practice context of TCM specialist nurses.

Given the diverse practice contexts in which TCM specialist nurses work, a theory‐informed lens is needed to clarify factors shaping EBP competence development and enactment. The Capability, Opportunity, Motivation, and Behavior (COM‐B) model conceptualizes behavior as arising from the interaction of capability (physical, psychological, and skill‐based factors), opportunity (external enabling factors), and motivation (cognitive processes influencing behavior) and has been used to guide understanding of behavior change and intervention design [21]. Although implementation science frameworks such as the Consolidated Framework for Implementation Research (CFIR) are well suited to examining organizational determinants and implementation processes [22], this study focused specifically on factors relevant to the development and enactment of EBP competence in practice. In this context, COM‐B was considered a suitable framework because it allows attention to individual capability, external opportunity, and motivational influences on practice. A systematic review further illustrates the utility of COM‐B for intervention development by mapping behavior change techniques used in interventions designed to strengthen healthcare professionals’ eHealth competency [23]. In nursing and midwifery research, the COM‐B model has been widely used to examine determinants of competence development (e.g., factors influencing core competence promotion among nurses and midwives) and to identify facilitators and barriers to EBP uptake in midwifery [24, 25].

Accordingly, with the COM‐B model as an interpretive tool, this study aimed to explore factors influencing EBP competence among TCM specialist nurses to inform training and organizational strategies that better enable evidence‐based TCM nursing.

## 2. Methods

### 2.1. Design

This was a qualitative study using semistructured interviews and theory‐informed thematic analysis to explore factors influencing the development and enactment of EBP competence among TCM specialist nurses.

### 2.2. Participants and Setting

Purposive sampling was used to recruit participants from multiple healthcare facilities in a province in Eastern China between April and August 2024. To capture variation in clinical contexts, participants were recruited from hospitals of different organizational types and grades within the Chinese hospital accreditation system. Eligible participants were in‐service registered nurses certified as TCM specialist nurses who were able to participate in a Mandarin interview, provide sufficiently detailed accounts of their work‐related experiences for qualitative inquiry, and give voluntary informed consent. Nurses who were not engaged in clinical practice or were on extended leave for more than 3 months were excluded.

Recruitment was led by the first author, a female postgraduate nursing student who also served as secretary of a specialist nurse training base. Recruitment notices were posted in WeChat, a widely used Chinese social media and messaging platform, in professional groups related to specialist nurse training and clinical practice. The first author had access to these groups through her role in training coordination and communication but did not hold managerial or evaluative authority over potential participants. Interested nurses contacted the research team voluntarily after reading the notice, and follow‐up phone communication occurred only after they had expressed interest and provided their contact details. Eligible participants then received further study information, and interviews were arranged at a mutually convenient time. Participation was entirely voluntary, and participants were informed that their decision to participate or decline would not affect their training, employment, or professional evaluation. Maximum variation purposive sampling was used across hospital types, hospital grades within the Chinese hospital accreditation system, clinical departments, seniority, and working roles (staff nurse or head nurse). Participant characteristics are summarized in Table 1. Sample size was guided by data saturation. Following Francis et al. [26], data collection continued until no new themes were identified in three consecutive interviews after analysis of at least 10 interviews. Data saturation was reached at the 15th interview, with two additional interviews conducted to confirm stabilization.

**TABLE 1:** Demographic and professional characteristics of 17 interviewees.

	Variables	*N*	%
Gender	Female	17	100

Age (years)	25–35	7	41.1
	36–45	8	47.0
	> 45	2	11.7

Education background	Associate degree	1	5.8
	Bachelor’s degree	12	70.5
	Master’s degree or above	4	23.5

Work experience (years)	5–10	2	11.7
	11–20	13	76.4
	> 20	2	11.7

Certification awarding level	Municipal	3	17.6
	Provincial	13	76.4
	National	1	5.8

Role	Staff nurse	13	76.4
	Head nurse	4	23.5

Hospital grade	Grade 3	9	52.9
	Grade 2	7	41.1
	Grade 1	1	5.8

Hospital type	Traditional Chinese medicine (TCM) hospital	12	70.5
	Integrative Chinese–Western medicine hospital	3	17.6
	General hospital (Western medicine–based)	2	11.7

Clinical department	Nephrology	3	17.6
	Oncology	3	17.6
	Intensive Care Unit	2	11.8
	Orthopedics	2	11.8
	Rehabilitation	2	11.8
	Endocrinology	2	11.8
	General surgery	2	11.8
	Gastroenterology	1	5.9

*Note:* Certification awarding level: refers to the administrative level of the certifying authority that awarded the TCM specialist nurse certification (municipal, provincial, or national). Hospital grade: refers to the level of the participant’s employing hospital within China’s hospital accreditation system (e.g., Grade 1, Grade 2, or Grade 3), rather than the accreditation rating within each grade. Grade 3 hospitals are upper‐level hospitals that typically provide comprehensive specialist and regional referral services. Head nurse: a nurse manager responsible for a ward or unit. Clinical department: the main clinical specialty area in which the participant was working at the time of interview.

Abbreviation: TCM = traditional Chinese medicine.

### 2.3. Data Collection

Semistructured, face‐to‐face interviews were used to collect data. The interview guide was developed based on the study aims, relevant literature on EBP competence, and barriers and facilitators influencing EBP, as well as sensitizing concepts drawn from the COM‐B model. The guide was reviewed within the research team for content relevance and clarity and was pilot‐tested with two TCM specialist nurses. Minor revisions were made to improve wording, question order, and the use of probes (see Table 2). These pilot interviews were not included in the final analysis. Interviews were conducted by the second and third authors (Chen Yu and Xu Wenji), both of whom had received formal training in qualitative research methods. No prior relationships existed between the interviewers and participants. Interviews were held in quiet, private locations to ensure confidentiality. All interviews were audio‐recorded, and field notes were taken to document contextual observations and nonverbal cues. Interviews were conducted in Mandarin, lasted approximately 30–45 min, and followed a flexible approach that allowed variation in question order and the use of follow‐up probes.

**TABLE 2:** Semistructured interview key questions.

Interview questions.
1. “What is your understanding of evidence‐based nursing (EBN) within your traditional Chinese medicine nursing (TCN) practice context, and how have you gained this knowledge?”
2. “How would you rate your current evidence‐based practice (EBP) competence? In which areas do you feel you need improvement?”
3. “Have you participated in any specific EBN training or practical implementation? If yes, how did these activities influence your competencies? What specific challenges have you encountered?”
4. “What are facilitators and barriers that influence your EBP competence?”
5. “What other factors should be considered when implementing EBP in traditional Chinese medicine nursing settings?”
6. “What are your suggestions for strengthening EBP competence?”
7. “What is your plan to enhance your EBP competence?”

### 2.4. Data Analysis

Interview recordings were transcribed verbatim as soon as feasible after each interview and checked against the audio recordings for accuracy before analysis. Transcripts were made available to participants for factual verification and subsequently imported into NVivo 14.0 [27] for data management and analysis.

Data were analyzed using theory‐informed thematic analysis, drawing on Braun and Clarke’ s six‐phase framework [28]. The Chinese transcripts were read repeatedly to achieve familiarity with the data, and initial codes were generated in an open manner, remaining attentive to meanings arising from participants’ accounts. Two researchers were involved in coding and theme development, with codes and emerging themes discussed iteratively and refined through analytic dialog within the research team. In the final analytic stage, themes were reviewed and interpreted in relation to the COM‐B model as a sensitizing and interpretive framework to clarify how capability, opportunity, and motivation‐related factors shaped the development and enactment of EBP competence. The coding and thematic structure were reviewed by the corresponding author, and final themes were confirmed through team consensus. Interviews were conducted and analyzed in Chinese to preserve semantic and contextual meaning. English translation was undertaken only for reporting preparation for illustrative quotations. Translated extracts were prepared by the research team and reviewed by the corresponding author against the original Chinese transcripts for conceptual equivalence.

### 2.5. Rigor

Credibility was enhanced by developing the interview guide from the study aims, relevant literature, and sensitizing concepts from the COM‐B model and by refining it through pilot testing. Data collection and analysis were iterative, with regular team discussions used to review codes, challenge interpretations, and refine themes. Dependability was supported through detailed documentation of coding, theme refinement, and team discussions. Confirmability was strengthened through the use of audio recordings, detailed field notes, and team‐based review of the coding and thematic structure. Researcher positioning and reflexivity were considered throughout data collection and analysis. The first author, who assisted with recruitment, was a postgraduate nursing student and secretary of a specialist nurse training base. Although she had no managerial or evaluative authority over participants, this role may have influenced access to participants and assumptions about specialist nurse development. To mitigate this risk, recruitment was conducted through voluntary responses to group postings, contact was initiated by interested nurses themselves, and participants were assured that their decision to participate would not affect their training, employment, or appraisal. The interviews were conducted by two researchers with qualitative training who had no prior relationships with participants. During analysis, the team engaged in regular reflexive discussions to examine how their clinical and academic backgrounds, including familiarity with EBP and TCM nursing, might shape coding and theme development. Initial coding remained open to meanings arising from the data, and the COM‐B model was used at the subsequent interpretive stage rather than as a rigid a priori coding structure.

### 2.6. Ethical Considerations

Ethical approval was obtained from the Ethics Committee of Jiangsu Province Hospital of Chinese Medicine (Approval No. 2024NL‐001‐02), and the study adhered to the Declaration of Helsinki. Written informed consent was obtained from all participants following an explanation of the study purpose and procedures, including voluntary withdrawal at any time without penalty. Audio files were securely stored, and participant identities were anonymized using coded identifiers.

## 3. Findings

Seventeen TCM specialist nurses participated in the study. Their demographic and professional characteristics are presented in Table 1. The mean age of the participants was 42 years (ranging from 30 to 52). All participants (*n* = 17) held at least a bachelor’s degree.

**FIGURE 1:**
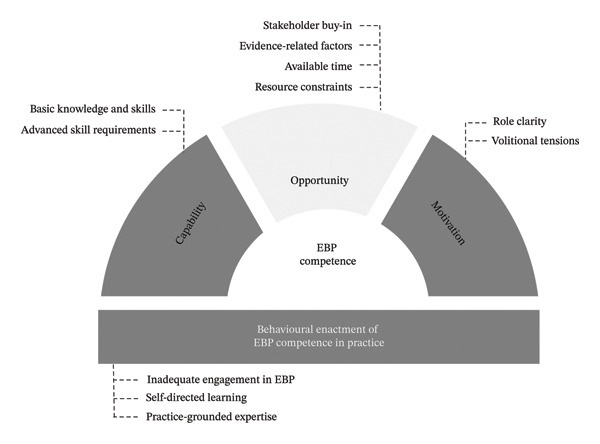
Thematic domains related to EBP competence, interpreted in relation to the COM‐B model, with behavioral enactment presented as the practical expression of competence in practice.

Participants described EBP as evidence‐informed clinical decision‐making that involves locating and appraising evidence and adapting it to real clinical situations (Participant 7). Some also questioned what should count as “evidence” in TCM nursing and how different sources (e.g., clinical experience, case reports, and expert opinion) should be weighted, while noting limited access to practice‐ready resources such as guidelines or consensus (Participant 15). Thematic analysis identified 11 subthemes across four overarching domains: capability‐related factors, opportunity‐related factors, motivation‐related factors, and behavioral enactment of EBP competence in practice. These domains were subsequently interpreted in relation to the COM‐B model (Figure 1).

### 3.1. Theme 1: Capability

#### 3.1.1. Basic Knowledge and Skills

Participants often described limited confidence in their EBP competence, which they related to gaps in foundational knowledge and skills.

*“Applying research findings to clinical practice inevitably involves statistics, such as data analysis, which is particularly challenging for me.” (Participant 12)*

*“I graduated from a secondary school… Basic skills like English have been unused for nearly 20 years, and my vocabulary has faded over time.” (Participant 11)*

Several participants emphasized that their proficiency in clinical knowledge and skills significantly influenced their ability to acquire and evaluate evidence, particularly in TCM nursing.

*“To solve clinical problems, one needs both classic TCM theories and extensive experience. Experienced nurses can apply research findings more effectively and are more likely to succeed.” (Participant 16)*

#### 3.1.2. Advanced Skill Requirements

Some statements highlighted that communication and coordination skills were crucial for effectively managing clinical work.

*“If you ask a patient whether they want to try a new therapy for symptom relief, they may think it doesn’t matter and simply refuse. I approach it from their perspective, communicating professionally and humanely to gain their cooperation and trust.” (Participant 14)*

Critical thinking was also emphasized, as participants reflected on the importance of assessing whether others’ experiences were supported by high‐level evidence and adapting them to real‐life scenarios.

*“Before implementing any (TCM nursing) therapies, I consult high-quality papers, gather evidence systematically, and adapt findings to real conditions. Simply copying others′ outcomes and practical experiences does not work in clinical settings.” (Participant 7)*

*“In the past, we blindly followed procedures, but now we critically evaluate them and actively seek supporting evidence.” (Participant 8)*

### 3.2. Theme 2: Opportunity

#### 3.2.1. Stakeholder Buy‐In

Participants described healthcare leaders, other healthcare practitioners, and patients as important influences on EBP competence. Many noted that despite receiving EBN training, a lack of support by stakeholders was a key barrier to their competency development.

*“Hospital leadership believes it is sufficient for doctors to generate revenue, and there is no need for nurses to further develop professionally.” (Participant 2)*

*“Our manager does not even set any expectations for us in terms of EBP competence.” (Participant 1)*

Conversely, some participants reported strong support from nurse managers, which facilitated their engagement with EBP.

*“The head nurse supports my efforts in implementing EBP and even arranges opportunities for me to conduct projects in the Nursing Clinic. Although I cannot manage all department tasks simultaneously, my performance evaluation and salary remain unaffected.” (Participant 2)*

Participants also noted that the attitudes of other healthcare professionals could influence whether evidence‐based initiatives were accepted in practice.

*“Doctors, technicians, and other healthcare practitioners are willing to support this initiative and actively refer patients to participate.” (Participant 14)*

*“During my shift, a postoperative patient experienced nausea and vomiting. I recommended intradermal needle therapy, which proved effective. However, the surgeon dismissed my suggestion outright, stating, ′I′m not interested and won′t complete the paperwork.′ This rejection, especially in front of the patient, made the situation quite uncomfortable.” (Participant 4)*

#### 3.2.2. Evidence‐Related Factors

Participants expressed concerns about the nature of evidence in TCM nursing, as it differs from the evidence hierarchy in conventional evidence‐based medicine.

*“What precisely qualifies as evidence in our practice? Can practical experience in TCM nursing serve as valid evidence? Are case studies and expert opinions acceptable, and how credible are they? These critical questions highlight the complexity and uncertainty inherent in evidence-based TCM nursing.” (Participant 15)*

*“Implementing EBP in TCM nursing remains challenging due to a significant shortage of robust evidence, including clear guidelines or expert consensus. Additionally, there is limited high-quality research specifically addressing TCM nursing, particularly within critical-care nursing, my area of expertise.” (Participant 13)*

#### 3.2.3. Available Time

Lack of time was another major barrier. Participants, already burdened with TCM nursing duties alongside routine responsibilities, struggled to find time for EBN learning. Married participants also faced additional domestic responsibilities.

*“I had planned to collect cases and analyze research data, but unexpected work demands disrupted my schedule.” (Participant 14)*

*“Work is too demanding, and family responsibilities of raising a child further limit the time available for learning.” (Participant 15)*

#### 3.2.4. Resource Constraints

Participants described resource constraints as another important barrier to EBP competence. Nurses from county‐level hospitals noted systemic limitations inherent in their institutional settings, describing them as difficult to change. Meanwhile, participants from tertiary hospitals reported intense internal competition for limited resources, despite having comparatively better infrastructure.

Participants from grassroots hospitals described rigid organizational environments that hindered innovation. *“In a small hospital, there are limitations beyond my control” (Participant 5, county-level hospital).* Resource constraints were also evident in tertiary hospitals, necessitating improvisation. *“Space is extremely limited—I don’t even have a dedicated treatment room. We had to create a makeshift area at the entrance using a screen and an examination bed from the duty room” (Participant 14, tertiary hospital).*

The absence of dedicated information platforms and interdisciplinary collaboration tools further impeded the implementation of EBP. One nurse from a tertiary hospital emphasized, *“We urgently need specialized platforms to facilitate collaboration with external institutions and integrate advanced technology for TCM nursing development, but internal competition poses significant challenges” (Participant 15, tertiary hospital).*

Another nurse similarly highlighted resource limitations, stating, *“Our proposal for a collaborative project with the university’s research team was rejected by the hospital due to budget constraints” (Participant 7, tertiary hospital).*

### 3.3. Theme 3: Motivation

#### 3.3.1. Role Clarity

Clearly defined professional roles and career pathways motivated some participants to enhance their EBP competence. One participant stated, *“Since I hold this role as a TCM specialist nurse, I must proactively take initiative” (Participant 14).* Another emphasized the benefits: *“Specialist nurses resolve complex patient issues, prompting us to seek evidence or consult senior experts. This process benefits both patients and nurses” (Participant 10).*

In contrast, unclear role expectations hindered others’ professional growth. *“My position as a TCM specialist nurse is equivalent to ordinary nurses, with no recognition or differentiation in professional achievements or research contributions” (Participant 6).* Another echoed similar frustrations, noting, *“We’re expected to excel in TCM nursing techniques, research, teaching, and evidence-based practice simultaneously, which is unrealistic given our ordinary capabilities” (Participant 7).*

#### 3.3.2. Volitional Tensions

Participants emphasized that internal motivation was a key driver in developing EBP competence.

*“Deep down, I aspire to enhance my EBN skills. The effort I put in brings me joy and satisfaction, motivating me to keep progressing” (Participant 10).*

*“When it comes to EBN in TCM nursing, I believe interest is fundamental. Without genuine interest, it′s unlikely one would actively engage in it” (Participant 1).*

Conversely, some participants described low motivation or reduced initiatives.

*“At 45, I just want to take things easier. Senior colleagues often advise focusing on reducing stress rather than taking on more responsibilities” (Participant 11).*

*“The main issue is my lack of initiative. If I were truly committed to learning, my competence could improve” (Participant 13).*

### 3.4. Theme 4: Behavioral Enactment in Practice

#### 3.4.1. Inadequate Engagement in EBP

Participants described limited and sometimes inconsistent engagement in EBP‐related activities. Many reported that EBN training was available but was often experienced as lecture‐based and difficult to translate into practice. They therefore emphasized the importance of practical learning opportunities that would allow them to apply methods to real clinical problems, receive feedback, and participate in actual evidence‐based projects.

*“We prefer hands-on courses where we can practice, receive feedback, and solve real problems. That’s how we truly grasp the methods” (Participant 4).*

*“The hospital conducts evidence-based training sessions regularly, yet the lecture content tends to fade from memory over time. It would be invaluable if the hospital could incorporate more EBP practicums, enabling us to learn through hands-on participation in real-world evidence-based projects.” (Participant 12)*

#### 3.4.2. Self‐Directed Learning

Participants recognized self‐directed learning as a key to developing EBP competence. *“Specialist nurses tend to think and act proactively, integrating evidence-based knowledge into clinical practice” (Participant 14).* Another reflected on the learning process: *“EBN promotes active thinking and learning. For instance, when I wondered why 10-minute cupping was recommended over 30 min, I searched for evidence and found it related to differences in efficacy and potential patient harm” (Participant 8).*

#### 3.4.3. Practice‐Grounded Expertise

Participants described active engagement in clinical practice as important for strengthening both theoretical understanding and technical proficiency.

*“Whenever I encounter complex cases, I thoroughly review both modern literature and classical medical texts. Solutions must be rooted in real clinical situations, not developed in isolation” (Participant 1).*

*“Practical engagement is the only way to truly develop EBN skills” (Participant 15).*

## 4. Discussion

To our knowledge, this is the first qualitative study to explore factors influencing EBP competence among TCM specialist nurses using the COM‐B model. The findings reveal that EBP competence is shaped by a combination of fundamental capabilities, external opportunities, and internal motivation and is expressed through how competence is enacted in practice. While several barriers identified here are consistent with those reported in broader nursing literature, this study also highlights contextual features of TCM nursing that may shape how EBP competence is understood, developed, and applied.

This study found that both basic and advanced competencies are essential for the development of EBP competence among TCM specialist nurses. Core skills such as literature retrieval, critical appraisal, and statistical literacy have been consistently identified as prerequisites for effective EBP engagement [29]. These findings are also consistent with Chang et al. [30], who emphasized that basic knowledge, communication skills, and EBN proficiency are integral to advancing nurses’ capabilities in TCM nursing contexts [30]. In addition to general EBN skills, professional expertise in TCM nursing knowledge and techniques appeared to support EBP competence. Nurses who are proficient in TCM nursing may be better equipped to manage complex or atypical clinical symptoms, thereby supporting service quality and patient safety [31]. Advanced competencies, including communication, coordination, and critical thinking, were closely related to EBN engagement, echoing findings from studies conducted in other nursing contexts [32]. Given nurses’ limited right of prescription in China, effective communication with patients and interdisciplinary teams is critical for evidence‐informed decision‐making. Critical thinking, in particular, plays a pivotal role in clinical judgment, problem identification, and the application of appropriate TCM interventions. However, studies in China have reported deficits in critical thinking among clinical nurses and nursing graduates [33, 34]. Rather than suggesting that TCM specialist nurses necessarily possess stronger critical thinking than other nursing groups, our findings indicate that participants viewed contextual judgment and reflective appraisal as central to the use of evidence in TCM nursing practice.

When judged against standards primarily designed for conventional biomedical trials (e.g., drug trials), some TCM nursing studies may appear to have methodological limitations and may therefore be classified as low‐level evidence [9, 31]. However, TCM nursing interventions are often complex and context‐dependent, and the evidence available to inform practice may be heterogeneous and not always translated into practice‐ready guidance [20]. Accordingly, evaluation criteria derived from conventional biomedical research may not always be directly applicable to TCM nursing interventions. In addition, TCM specialist nurses need to draw on both modern research and classical knowledge to synthesize the best available evidence for practice, which may support clinical reasoning and help make more robust, contextually relevant nursing decisions.

The findings related to opportunity suggest that EBP competence among TCM specialist nurses is strongly shaped by contextual conditions. Patients, as principal stakeholders in the EBN process, significantly influence the adoption of optimal evidence, thereby impacting the EBP competence of TCM specialist nurses. A systematic review [35] incorporating 39 studies affirmed that patient comprehension and cooperation facilitate the activation of EBN in TCM nursing practice. The engagement of stakeholders beyond patients, including physicians, technicians, and healthcare leaders, has similarly been shown to affect nurses’ EBP competence [15]. Notably, the support and buy‐in of stakeholders, particularly nurse managers or administrators, may play crucial roles in shaping nurses’ EBP competence, as emphasized in previous studies [25]. Elwy et al. [36] also highlighted the importance of stakeholder buy‐in for evidence application and proposed a three‐step approach to identifying stakeholders and obtaining their support, which may offer useful guidance for strengthening EBP competence among TCM specialist nurses.

Our findings further suggest that evidence‐related challenges in TCM nursing not only are limited to evidence availability but also involve uncertainty about what should count as evidence and how different forms of evidence should be applied in practice. This is consistent with previous research indicating that reliance on practical wisdom or tradition, in the absence of robust and practice‐ready evidence, may constrain EBP competence development in Chinese medicine nursing context [3]. Although efforts have been made to establish grading systems for TCM nursing evidence, their application in clinical practice remains inconsistent [3]. These findings suggest a need for tailored training approaches that address this distinctive challenge. EBN emphasizes standardization through optimal evidence application, whereas TCM nursing adopts a holistic approach balancing standardization with personalization via syndrome‐differentiated care. This tension may represent an important challenge in advancing evidence‐informed TCM nursing. As advocated in a review Fung & Yin, [17], evidence‐based principles may need to be adapted to TCM rather than rigidly imposed, allowing flexibility that accounts for the unique characteristics of TCM. Building on our findings that participants reported uncertainty about what should count as “evidence” and limited access to practice‐ready guidance, future efforts should focus on translating heterogeneous evidence into usable forms for clinical care. For example, locally agreed, practice‐ready resources that explicitly incorporate syndrome differentiation and clarify how research evidence, clinical expertise, and patient preferences may be integrated could make evidence use more transparent and feasible in routine TCM nursing. Such approaches may help reconcile standardization demands with contextual fit and support more feasible EBN uptake in TCM nursing.

The constraints of available time and institutional software and hardware resources likewise surface as complementary secondary themes within the opportunity theme, aligning with previous research outcomes in Shanghai, China [16], the United States [37], and Spain [13]. Most specialist nurses work part‐time [38], requiring them to shoulder additional duties, including scientific research, teaching, and management, which aligns with this study’s findings. Many also face family caregiving responsibilities, further limiting their availability for continuous professional development. While larger tertiary hospitals may theoretically offer more EBN resources, participants in this study reported that intense internal competition and bureaucratic hurdles often restricted actual access to those resources. This contrasts with the broader assumption that tertiary institutions are necessarily more enabling environments for EBN [15, 39–42]. Taken together, these findings suggest that improving EBP competence among TCM specialist nurses may require not only stronger educational support and stakeholder engagement but also organizational conditions such as protected time, more equitable resource access, and reduced workload fragmentation.

We also found that role clarity affects participants’ EBP competence. Previous studies [38] have shown that role ambiguity and unclear responsibilities can make it difficult for specialist nurses to focus on their work, whereas clearer work differentiation may promote nurses’ use of EBP. This study further highlights the relevance of role clarity to EBP competence. In addition to providing evidence‐based TCM nursing care for patients, TCM specialist nurses are often expected to undertake responsibilities in education and administration [30]. Although some hospitals launched Chinese nursing clinics, the post settings, qualification management, and matching training plans still lack unified norms [8, 43], which undermine the continuity and stability of TCM specialist nurses’ development and, in turn, impede their EBP competence. These findings suggest that clearer role descriptions and more coherent role arrangements may help support tailored training and long‐term professional development.

Sustained engagement in clinical practice and self‐directed learning appeared to be important ways through which TCM specialist nurses strengthened their EBP competence, complementing the capability‐related subtheme of basic knowledge and skills. Although knowledge facilitation is the initial step for Chinese nurses in EBP [8], this study suggests that mastering fundamental knowledge and skills alone may be inadequate to enhance the EBP competence of TCM specialist nurses. Continuous learning and deeper engagement in both TCM nursing practice and EBN activities may therefore be important. This also resonates with another subtheme identified in this study that insufficient participation in EBP and the limitations of current training approaches may constrain the EBP competence of Chinese specialist nurses. A Delphi study on Chinese nurses’ EBN capabilities pointed out that “application” domain items are particularly important for EBN [12], whereas many current EBP education interventions continue to focus primarily on evidence retrieval and critical appraisal, with comparatively less attention to the evidence translation in clinical practice [44]. These findings suggest that greater emphasis on evidence translation in training may be warranted. Developing and implementing tailored EBP training for TCM specialist nurses may therefore help strengthen their ability to apply evidence in clinical work.

Taken together, these findings suggest that EBP competence among TCM specialist nurses is shaped by interacting capability, opportunity, and motivation‐related factors and is expressed through how competence is enacted in practice. Strengthening EBP competence in this group may therefore require not only capability‐building through training but also clearer role expectations, practice‐ready evidence resources, and supportive organizational conditions. These implications should, however, be interpreted cautiously, given that this study was based on a qualitative sample from a single regional context.

## 5. Limitations

The study was conducted in a single regional context, and all participants were female, reflecting the limited availability of male TCM specialist nurses at the time of recruitment. These factors may limit the transferability of the findings, and future research should aim to include more diverse samples across broader settings.

## 6. Conclusion

This study is among the first qualitative study to explore determinants of EBP competence among TCM specialist nurses. The findings extend the understanding of how capability, opportunity, and motivation‐related factors shape EBP competence among TCM specialist nurses. Further studies including male TCM specialist nurses and conducted across multiple regions or institutions are warranted to improve the transferability of the findings.

**Nomenclature**
Evidence‐based practice (EBP): Evidence‐based practice (EBP) is an approach to the delivery of healthcare that integrates the best evidence from well‐designed studies (i.e., external evidence) and integrates it with a patient’s preferences and values and a clinician’s expertise, which includes internal evidence gathered from patient data
Evidence‐based nursing (EBN): Evidence‐based nursing (EBN) is an integration of the best evidence available, nursing expertise, and the values and preferences of the individuals, families, and communities who are served
Specialist nurse (SN): Specialist nurse (SN) is a nurse with advanced nursing knowledge and skills, educated beyond the level of a generalist or specialized nurse, in making complex decisions in a clinical specialty and utilizing a systems approach to influence optimal care in healthcare organizations
Traditional Chinese medicine nursing (TCM nursing): Traditional Chinese medicine (TCM) nursing is a practical science that is guided by theories of traditional Chinese medicine, combining prevention, healthcare, and rehabilitation, to care for the old, the weak, the young, and the handicapped, and applying specific nursing techniques to promote the health of the people

## Author Contributions

Study design: Yan Li and Shizheng Du.

Data collection: Yu Chen, Wenji Xu, and Jie Sun.

Data analysis: Yan Li, Yu Chen, Wenji Xu, Jie Sun, and Shizheng Du.

Study supervision: Shizheng Du.

Manuscript writing: Yan Li, Shizheng Du.

Critical revisions for important intellectual content: Yan Li and Shizheng Du.

## Funding

This work was supported by the Postgraduate Research & Practice Innovation Program of Jiangsu Province (Grant Number: SJCX24_0816) and the Researcher‐Initiated Project of the Jiangsu Province Hospital of Chinese Medicine (Grant Number: YJZ202366).

## Disclosure

The funding bodies had no role in the design, implementation, writing, or submission for publication of the study.

## Ethics Statement

This study received approval from the Ethics Board of Jiangsu Province Hospital of Chinese Medicine before its commencement (application number: 2024NL‐001‐02) and strictly adhered to the ethical principles outlined in the Declaration of Helsinki.

## Conflicts of Interest

The authors declare no conflicts of interest.

## Supporting Information

Additional supporting information can be found online in the Supporting Information section.

## Supporting information


**Supporting Information** Consolidated criteria for reporting qualitative studies (COREQ) checklist.

## Data Availability

The datasets used and analyzed during the current study are available from the corresponding author upon reasonable request.
